# Effect of Ceramic Thickness and Technician Variability on the Shade Duplication of Dental Ceramo-Metallic Restorations

**DOI:** 10.3390/jfb15010012

**Published:** 2023-12-28

**Authors:** Rasha M. Abdelraouf, Taheya A. Moussa, Tamer M. Hamdy, Reem A. Abuhaimed, Alanoud M. Alotaibi, Carlos A. Jurado, Abdulaziz Alhotan, Bushra Alhelal, Nour A. Habib

**Affiliations:** 1 Biomaterials Department, Faculty of Dentistry, Cairo University, Cairo 11553, Egypt; rasha.abdelraouf@dentistry.cu.edu.eg (R.M.A.); taheya.mosaa@dentistry.cu.edu.eg (T.A.M.); nour.habib@dentistry.cu.edu.eg (N.A.H.); 2 Restorative and Dental Materials Department, Oral and Dental Research Institute, National Research Centre (NRC), El Bohouth St., Dokki, Giza 12622, Egypt; tm.hamdy@nrc.sci.eg; 3 Dental Interns, College of Dentistry, King Saud University, P.O. Box 60169, Riyadh 11545, Saudi Arabia; reemiabdulaziz16@gmail.com (R.A.A.); alanoud_alotaibi@hotmail.com (A.M.A.); 438200128@student.ksu.edu.sa (B.A.); 4 Department of Prosthodontics, University of Iowa College of Dentistry and Dental Clinics, Iowa City, IA 52242, USA; carlos-jurado@uiowa.edu; 5 Department of Dental Health, College of Applied Medical Sciences, King Saud University, P.O. Box 10219, Riyadh 12372, Saudi Arabia

**Keywords:** ceramic thickness, technician variability, shade duplication, ceramics, ceramo-metallic restorations, metal ceramic

## Abstract

Ceramic thickness and technicians’ manipulative variables are critical factors affecting the resultant shade of dental ceramo-metallic restorations. This study investigated the effect of the following variables on shade duplication of ceramo-metallic specimens: (a) ceramic thickness; (b) differences between several technicians (inter-technician variability); and (c) the ability of each technician to repeat the resultant shade (intra-technician variability). Ninety ceramo-metallic specimens were prepared and divided into three main groups (*n* = 30/gp) according to the different technicians who built up the veneering ceramic of the specimens. Each group was further subdivided into three subgroups (*n* = 10/subgroup) according to the thickness of the ceramic (1, 1.5, and 2 mm built over a 0.5 mm-thick metal substructure). Three different technicians were asked to follow the same protocol as regards the same ceramic batch (Shade 3M2, Vita VM13, Zahnfabrik, Germany), firing temperature, and number of firing cycles. Meanwhile, each technician followed his own protocol with regard to other ceramic manipulative variables. The duplicated shades of the specimens were investigated using the Vita Easyshade spectrophotometer by using the verify shade mode. Color difference (∆E) values were calculated between the target shade (3M2) and the duplicated shades of the specimens automatically by the Vita Easyshade spectrophotometer (Vita, Zahnfabrik, Germany). The effect of ceramic thickness and inter- and intra-technician variability on the duplication of the target shade was investigated. The results showed that the effect of ceramic thickness on the duplicated shades depended on inter-technician variability. High inter-technician variability (∆E = 2–6.4) was noticed in contrast to low intra-technician variability (∆E = 0.2–1.5). It could be concluded that proper shade-duplication of ceramo-metallic restoration was a cumulative technique intimately related to manipulative variables and ceramic thickness.

## 1. Introduction

After almost 40 years of successful use, metal-ceramic restorations continue to be a common indirect restorative system [1]. A skilled technician can create a metal ceramic crown as stunning as an all-ceramic restoration [1]. Dental ceramics are brittle and easy to fracture. In order to counteract this weakness, ceramics are typically reinforced and supported with metal to create a metal ceramic restoration [2]. 

There have been developments in dental ceramics that extend to all-ceramic restorations, which become the material of choice when aesthetics are crucial since they successfully mimic the visual characteristics of the tooth substance [2,3,4]. It combines strength and esthetics for acceptable and durable dental restorations [5]. There is also development in dental ceramic fabrication techniques, which include conventional condensation methods, heat pressing, subtractive manufacturing by computer-aided design, computer-aided manufacturing (CAD-CAM), and additive manufacturing by 3-D printing. It should be noted that the conventional condensation technique could be used to veneer ceramic cores fabricated by other methods, such as CAD-CAM or heat-pressing [6].

However, even with the introduction of all-ceramic restorations, ceramo-metallic restorations are still frequently used in dentistry. In addition, there are situations where ceramo-metallic restoration is recommended, such as severe bruxism, moderate gingival inflammation, high caries rates, and poor oral hygiene [7]. Shade matching in ceramo-metallic restorations is difficult because of the metal substructure [5]. In addition, the ceramic veneer overlying the metal substructure is usually built by the conventional condensation method, which mainly depends on the technician’s manipulative variables, where the porcelain powders are mixed with liquid and compacted over the metal substructure. The ceramic is built in layers, mainly opaque, dentin, and enamel. 

Various ceramic thicknesses can be employed based on the desired restoration type and amount of tooth reduction [5]. Evaluating the influence of material thickness on the optical features is crucial for enhancing the aesthetic outcome. Previous research assessed visually the influence of three ceramic thicknesses (0.5, 1, and 1.5 mm) on the resultant color. Thicker ceramic specimens gave better shade [8]. On the other hand, another study veneered twenty metallic examples of pure titanium with ceramics. The specimens were divided into four groups according to the ceramic thickness (0.5 mm, 1 mm, 1.5 mm, and 2 mm), with *n* = 5/group. A spectrophotometer was used to determine the color parameters [9]. Thicker specimens were darker than thinner ones [10]. There is a controversy in the literature about the exact effect of ceramic thickness on achieving the desired shade. This needs further study, especially in ceramo-metallic restorations, due to the challenge of masking the color of the metallic framework.

There is another factor that could affect the resultant shade of ceramo-metallic restorations. Proper shade reproduction of dental ceramo-metallic restorations depends on proper shade duplication by the dental technician. The fabrication of most ceramic restorations involved building different layers. These complex procedures of ceramic building influence the color of the finished restoration. Thus, producing a restoration with proper color matching represented a real challenge [11]. Technicians’ manipulative variables could affect the color of the veneering ceramic [3,6]. A previous study examined the variations in color reproduction for metal-ceramic crowns made by various dental laboratories. Five technicians created fifty metal-ceramic crowns. Each technician fabricated ten ceramo-metallic crowns of the same shade. The choice of ceramic type and construction method was left up to the technicians. A colorimeter was used to measure the color variations between the target shades and the crowns. Surprisingly, most crowns made by these technicians had unacceptable shades [12]. Aljamhan A.S. et al. (2022) investigated the color matching of dental ceramo-metallic specimens fabricated by different laboratories using three common shades. Ceramo-metallic discs were fabricated by six laboratories, with 30 specimens per laboratory divided into 10 for each shade (A1, B1, and C1). The results showed significant color differences among the same shades duplicated by various laboratories [13].

The objectives of the current study are to investigate the effect of ceramic thickness, inter-technician variability (difference between several technicians), and intra-technician variability (difference within the same technician) on shade duplication of ceramo-metallic specimens. Color matching was assessed instrumentally by calculating the color difference (∆E) values between the target shade (3M2) and the duplicated shades of the specimens. The null hypothesis stated that the ceramic thickness, inter-technician variability (difference between several technicians), and intra-technician variability (difference within the same technician) would not affect the shade duplication of ceramo-metallic specimens.

## 2. Materials and Methods

The commercial materials used in this study are represented in Table 1.

### 2.1. Study Design

**Figure 1:**
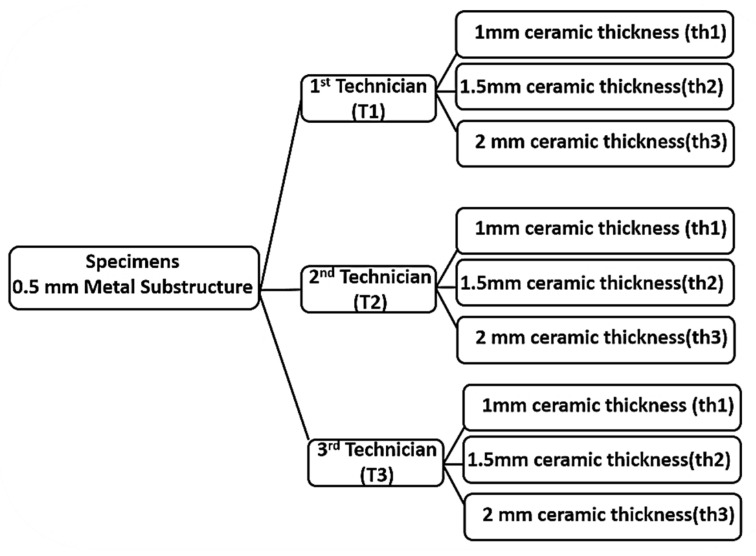
Flowchart of this study design.

Ninety ceramo-metallic specimens were prepared and divided into three main groups (*n* = 30/per group) according to the different technicians who built up the veneering ceramic of the specimens. Each group was further subdivided into three subgroups (*n* = 10/subgroup) according to the thickness of the ceramic (1, 1.5, and 2 mm built over a 0.5 mm thick metal substructure), as shown in Figure 1.

The sample size calculation was based on previously reported research [13]. With an alpha level of 0.05 and a power of 80%, a sample size per group of five test specimens was determined using G*Power software version 3.1.9.7 (Heinrich Heine University Duesseldorf, Duesseldorf, Germany). The analysis was carried out with an effect size of 0.8 and a level of significance of *p* < 0.05. Each technician should provide 10 samples in each thickness; the total samples from all groups were 90 (three thicknesses by three technicians); *N* = 90. 

### 2.2. Specimens’ Fabrication

#### 2.2.1. Mold Construction

**Figure 2:**
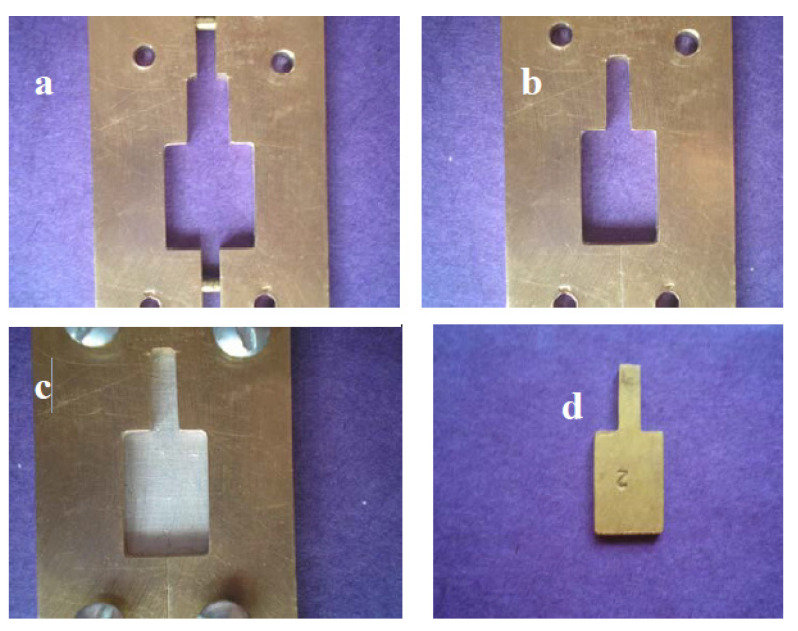
Split metallic copper mold: (**a**) Split mold (**b**) Closed mold (**c**) 4 screws and flat metallic sheet (**d**) One of the metallic templates.

A split metallic copper mold was designed and constructed (Figure 2). It had two parts attached to each other by means of pins and holes (Figure 2a,b). Moreover, there were four screws at the corners to fix that mold to a flat metallic sheet (Figure 2c). The mold had a rectangular central hollow of dimensions 15 mm × 10 mm with a thickness of 5 mm. In the middle part of the short side (10 mm), an extension of that hollow was constructed with dimensions of 10 mm × 3 mm to safely handle the specimens during ceramic building. 

The variation of the thickness in the mold had been adjusted with the help of extra-metallic templates (Figure 2d). An extra-metallic template of 4.5 mm thickness was used to build the wax pattern of 0.5 mm thickness for the metallic substructure. Other extra-metallic templates with thicknesses of 3.5, 3, and 2.5 mm when placed in the 5 mm thickness mold provided a space of 1.5, 2, and 2.5 mm, respectively. Thus, when the metallic substructures of the specimens (0.5 mm thickness) were placed over these metallic templates, 1, 1.5, and 2 mm were left for the veneering ceramic. 

#### 2.2.2. Metal Substructure Fabrication

##### Wax Pattern

After painting the mold with a thin layer of separating medium, softened pink wax (Cavex, Haarlem, Holland) was adapted to fill the mold (0.5 mm thickness). To achieve optimum smoothness for the wax pattern, flat glass plates were placed above the mold. After hardening of the wax, the mold was then split, and the wax pattern was retrieved out of the mold.

##### Casting Procedure

Wax sprues (thickness 2.5 mm × 10 mm length) (Renfant, Hilzingen, Germany) were attached to the handle of the wax patterns and then were attached to the crucible former of the casting ring. Graphite-free phosphate-bonded investment (Bellavest SH, Bego, Goldschlägerei Wilh. Herbst GmbH & Co., Bremen, Germany) was mixed according to the manufacturer’s instructions. One hour was allowed for the investment to harden. The manufacturer’s directions for casting the alloy were followed. The investment was left for bench cooling for two hours before devesting. 

##### Finishing and Sandblasting the Metallic Substructure

After devesting, the sprues were removed by a disc stone 0.7 mm thick. The specimens were finished with a fine, cross-cut tungsten carbide bur. The thickness of the metal surface was checked by a graduated caliper (Mitutoyo, Tokyo, Japan) at 9 points. Then, each substructure was sandblasted with 125 µm aluminum oxide at a pressure of 2 bars. 

##### Construction of Veneering Ceramic (Shade 3M2)

The basic layering technique of ceramics (wash opaque, opaque, base dentin, enamel) was followed in constructing the veneering ceramic of the specimens according to the manufacturer’s instructions. The wash opaque powder was mixed with the opaque fluid to a thin, watery consistency and was applied to the clean, dry metal substructures using a ceramic application brush. Specimens were fired as recommended by the manufacturer in a ceramic furnace (Vita Vacumat 40T, Vita Zahnfabrik, Bad Säckingen, Germany). 

**Figure 3:**
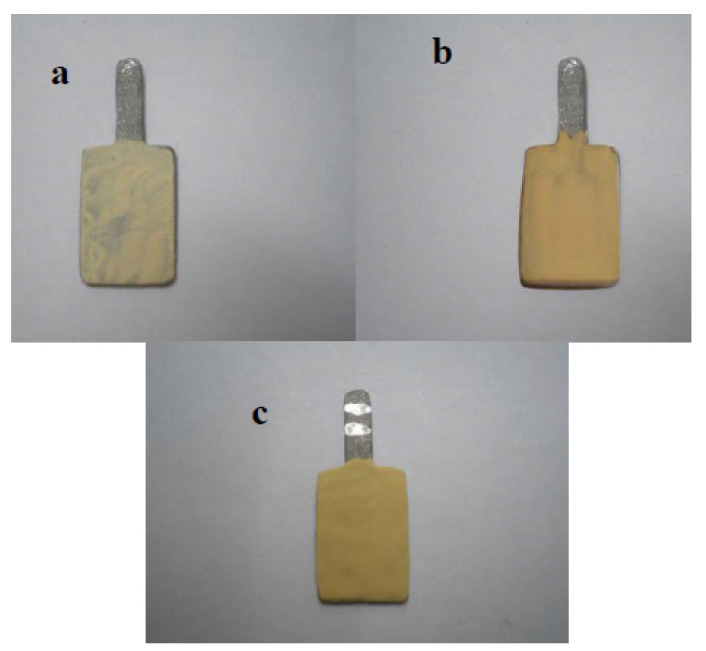
Wash the opaque layer built by (**a**) Technician 1 (T1), (**b**) Technician 2 (T2), and (**c**) Technician 3 (T3).

The three different technicians were asked to follow the same protocol regarding the same ceramic batch, firing temperature, and number of firing cycles. Because there was no standardized manufacturer instruction, each technician followed his own protocol regarding other ceramic manipulative variables. These included powder/liquid ratios, the type of spatula used for mixing, the consistency of the mix, the degree of compaction, and the thickness of each ceramic layer. Figure 3 shows the difference in the wash opaque layer built by Technician 1 (T1), Technician 2 (T2), and Technician 3 (T3).

The opaque powder was mixed with the opaque fluid to give a creamy consistency and was applied to the metal substructures upon firing. The specimens were inserted in the ceramic furnace and fired according to the manufacturer’s instructions.

**Figure 4:**
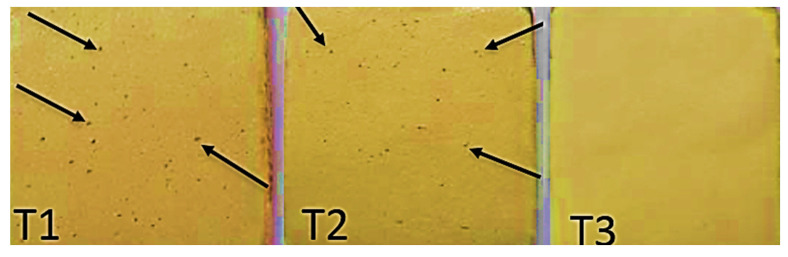
Opaque layers were built by the three technicians; arrows indicate porosities within the ceramics prepared by Technicians T1 and T2.

After firing the opaque layer, porosities were observed within the ceramics prepared by Technicians T1 and T2. On the other hand, no obvious porosities were detected in specimens prepared by T3 (Figure 4).

The thickness of the specimen with fired opaque layers was measured by the graduated caliper at 9 points (0.1–0.4 ± 0.05 mm). The thickness of the opaque layers by T1 and T2 was 0.1 ± 0.05 mm, while by T3 it was thicker, 0.4 ± 0.05 mm.

**Figure 5:**
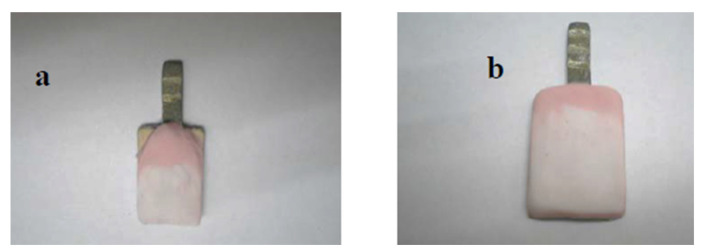
Dentin and enamel porcelain before firing (**a**) During building (**b**) After building.

Then, the previous substructures were inserted in the mold and adjusted by the metallic templates to build different ceramic thicknesses (1.5 mm for the construction of 1 mm ceramic thickness, 2 mm for the construction of 1.5 mm ceramic thickness, and 2.5 mm for the construction of 2 mm ceramic thickness). The base dentin powder was mixed with the modeling liquid to a creamy consistency and applied to these specimens. Clean tissue was used to absorb excess liquid, helping with the compaction of the ceramic powder. Enamel powder was mixed in the same way as base dentin powder. Several small portions of enamel were applied (Figure 5). To compensate for firing shrinkage, the size was made larger. The mixing and firing were conducted as previously mentioned. 

**Figure 6:**
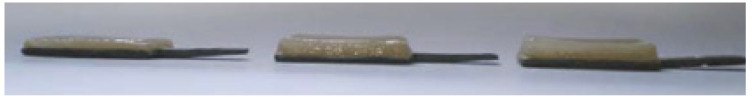
Specimens with 3 different ceramic thicknesses (1, 1.5, and 2 mm).

Prior to glazing, the entire surfaces of the specimens were ground by diamond bur evenly after they had been checked (in the mold and by a graduated caliper). For glazing, the surface of each specimen was coated with glaze material and fired as recommended. The veneering ceramic was built by each technician in three different thicknesses (1, 1.5, and 2 mm), as shown in Figure 6. The ceramic thicknesses of 1, 1.5, and 2 mm were denoted by th1, th2, and th3, respectively. 

### 2.3. Color Analysis of Specimens

**Figure 7:**
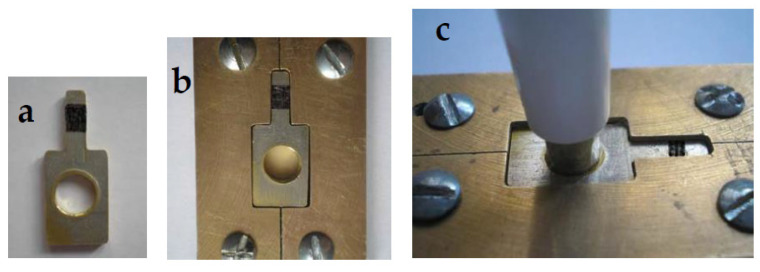
Assessing the shade of the specimens: (**a**) Template with circular hole; (**b**) Template over the specimen placed in the mold; (**c**) Spectrophotometer tip passing the hole.

The duplication of the target shade was investigated using a Vita Easyshade spectrophotometer in “Verify Restoration” mode. Shade 3M2 was chosen to be the target shade to assess the duplicated shade. The tip of the probe was adjusted to measure the middle third of the specimens. To standardize the measured area in all specimens, a special template with a circular hole was placed above the specimens inserted in the mold. The tip of the probe passed through its hole (Figure 7), and the handpiece switch was pressed.

The calculation of the color difference (∆E) between the duplicated shades of the specimens and the target shade (3M2) was performed automatically with the Vita Easyshade spectrophotometer (Vita, Vita ZahnFabrik, Bad Säckingen, Germany). The color parameters of the duplicated specimens were compared to the standard 3M2 parameters, which were input data installed within the device and used as a reference. 

The Vita Easyshade spectrophotometer had fiber optics arranged in a specific pattern. Some fiber optics illuminated the specimens, while others received the reflected light. The reflected light from the specimens was analyzed by the device software, which calculated the color parameters of the specimens and the color difference (∆E) compared to the target shade selected by the operator (shade 3M2 in this study).

The resultant ∆E values were compared to the perceptibility threshold (initial color difference visually detected; ∆E = 1.2) and acceptability threshold (more than this value is considered unaccepted; ∆E = 2.7) [14].

The effect of ceramic thickness and inter-technician variability (difference between several technicians) on the resultant shade was investigated by calculating the color difference (∆E) values between the target shade (3M2) and the duplicated shades of the specimens. In addition, intra-technician variability (difference within the same technician) was also calculated to assess the ability of each technician to repeat the resultant shade in each ceramic thickness ten times.

### 2.4. Statistical Analysis

A two-way ANOVA (analysis of variance) was used to test the difference between ceramic thicknesses, the variation between technicians (inter-technician variability), and their interaction. A post hoc test was performed to find which pairs significantly differed. Successive subtraction was used to calculate the difference in results within the same technician for the same ceramic thickness (intra-technician variability). The significance level was set at *p* ≤ 0.05. Statistical analysis was performed with SPSS 16.0^®^ (Statistical Package for Social Science, SPSS, Inc., Chicago, IL, USA) for Windows.

## 3. Results

### 3.1. Effect of Ceramic Thickness and Inter-Technicaian Variability on the Duplicated Shade

**Figure 8:**
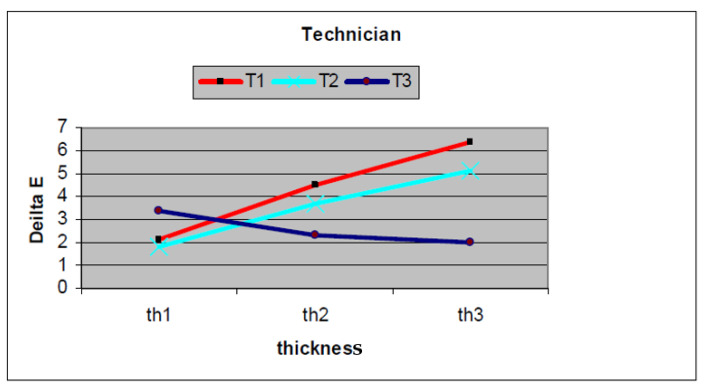
Effect of ceramic thickness on the shade duplication of ceramo-metallic specimens between different technicians.

There was a significant difference between the mean ∆E values among the different ceramic thicknesses (*p* = 0.00001) as well as between various technicians (*p* = 0.00001), as shown in Table 2 and Figure 8. It also showed that there was a significant interaction existing between the ceramic thickness and the technician (*p* = 0.00001). 

The ceramic thickness had a significant effect on the resultant shade (*p* = 0.00001). In Technicians 1 and 2, the least ceramic thickness (1 mm) had the least color difference, while the thickest ceramic thickness (2 mm) had the highest color difference. On the contrary, the opposite occurred in Technician 3, where the thickest ceramic thickness (2 mm) had the least color difference, while the least ceramic thickness (1 mm) had the highest color difference. 

Regarding inter-technician variability, technician 3 (T3) was significantly different (*p* = 0.00001) than Technicians 1 and 2, while no significant difference was detected between Technicians 1 and 2 (*p* = 0.3).

All these values exceeded the perceptibility threshold (∆E = 1.2) and, in some cases, exceeded the acceptability threshold (∆E = 2.7).

### 3.2. Effect of Intra-Technician Variability on the Shade Duplication of Ceramo-Metallic Specimens

Regarding intra-technician variability, no significant differences were detected, as shown in Table 3. These resultant color differences were lower than the perceptibility threshold (∆E = 1.2) or just above it. 

## 4. Discussion

Esthetics are one of the dental patients’ prime concerns. Direct tooth-colored restorations made of resin composite have become very common [15]. However, due to the relatively lower mechanical properties of resin composite material, indirect restorations are mainly all-ceramic-based or ceramo-metallic-based.

Metal-ceramic restorations are still considered one of the most commonly used fixed restorations, even with the introduction of all-ceramic restorations [1]. As regards patient selection sensitivity, several studies of all-ceramic crowns have exclusion criteria for patients with severe bruxism, moderate gingival inflammation, high caries rates, and poor oral hygiene. Brittle fracture liability is more critical with some types of all-ceramic versus metal-ceramic restorations. In addition, metal-ceramic crowns have a relatively low cost and have been documented with 94% success rates over 10 years [16]. In addition, the color contrast between the metal and the ceramic veneer might aid in displaying the manipulative variables between the technicians. Thus, shade duplication of metal-ceramic specimens was more concerning than all-ceramic in this study. 

Matching the color properties of natural dentition with artificial restoration is quite challenging in dental practice. For metal ceramic restorations to be clinically successful, color matching is crucial. Ceramic thickness is a critical factor in shade duplication that could affect the optical characteristics of the ceramic [10]. Proper color reproduction depends not only on the proper shade selection by the dentist but also on the proper shade duplication by the technician [17,18,19]. Thus, even if the proper color was selected, the use of this information by the dental technician to fabricate ceramic restorations would not guarantee a predictable color for this restoration. Several factors interfered with the shade duplication, such as inter-technician variability (difference between several technicians) and intra-technician variability (difference within the same technician). Therefore, this study investigated the effect of technician variability (inter and intra) as well as different ceramic thicknesses on shade duplication of ceramo-metallic specimens.

In this research, a split mold was used to allow easy retrieval of the wax patterns. Rectangular specimens were constructed because the rectangle is one of the most uniform geometrical patterns, resembling the outline of the labial surface of the upper central incisor tooth [20,21]. Moreover, it is a reproducible shape. A handle was constructed for each rectangular specimen to demarcate its cervical portion, “the neck”. Wax sprue was attached to this handle to avoid any distortion of the rectangular part, and it assisted in safely handling the specimens during ceramic construction [20]. 

Wax patterns were built from pink wax rather than blue inlay wax, as it showed better results in this study. Pink wax being already flat, less stress was induced in the material during shaping into a metallic mold, thus minimizing the distortion of the wax pattern. Moreover, pink wax showed low adherence to the metallic mold and low liability for internal defects, marginal excess, or deficient material. It was also time-saving during modeling in large areas [21]. 

Vita VM^®^ 13 was selected as a veneering ceramic because it offers proper handling characteristics, a homogeneous distribution of both crystal and glass phases, and clinical wear characteristics that mimic those of enamel. The thickness of the specimens’ metal substructures was 0.5 mm, nearly resembling the clinically relevant thickness of the metal in metal-ceramic restorations. While the ceramic thicknesses in this study were 1, 1.5, and 2 mm, this thickness range was recommended by the manufacturer, who claimed that veneering ceramic thickness under 0.8 mm was considered insufficient and above 2 mm, excessive tooth reduction was required. 

The metal substructures were cast by one technician to standardize all casting procedures [16]. Ceramic shade 3M2 was used in this part of this study, as it represents the mean shade in the Vitapan 3D-Master shade guide and one of the most common shades [22]. Manipulative steps performed during the building of the ceramic of the labial surface of the upper central incisor crowns were followed in this part of this study. This offered the advantage of being more informative in assessing the actual ceramic building procedure. Meanwhile, being flattened surface specimens, the instrumental color measurements were not affected by any curvature. 

To automatically quantify the color difference (∆E) between the target shade (3M2) and the duplicated shade of the specimens, a Vita Easyshade spectrophotometer was used [18,19]. The development of instrumental methods for color matching has been established to overcome the weaknesses of visual methods and offer impartial color determination. Spectrophotometers, digital cameras, and intraoral scanners (IOSs) are examples of these devices. A spectrophotometer can determine the color and produce CIELab values by evaluating the object’s spectral reflectance or transmittance [23]. The idea of color difference (ΔE) was proposed by CIE and correlated clinically to describe the acceptability and perceptibility of the duplicated shade compared to the desired shade (target/reference). Acceptability denotes acceptance of the restoration color, whereas perceptibility is connected to the ability to distinguish between the color of the restoration and the neighboring tooth [24]. 

The null hypothesis was rejected as there was a significant difference between the mean ∆E values among the different ceramic thicknesses as well as between various technicians.

Concerning the effect of ceramic thickness and inter-technician variability, the color difference (∆E) values ranged from 2 to 6.4. This means that the duplicated shades of the specimens were visually perceptible compared to the target shade (exceeding the perceptibility threshold; ∆E = 1.2) and, in some cases, unacceptable (exceeding the acceptability threshold; ∆E = 2.7), with a significant difference among technicians. This may be attributed to the absence of standardized technical instructions, which led to improper manipulative procedures. Metallic spatulas, rather than glass mixing rods, were used by the technicians. This may lead to their abrasion during ceramic mixing, producing metal debris that could discolor the fired ceramic restoration and increase ∆E. It was noticed that different mix consistency was used by three technicians. This may be attributed to the absence of measures supplied by the manufacturer to ensure a standardized powder/liquid mixing ratio.

The powder/liquid ratio may influence the thickness of the resultant layer [25]. This may be critical, especially for the first layer of the ceramic (washed opaque), which must be applied in a thin layer for both proper thickness and proper wetting. In addition, improper wetting of the metallic substructure by a thick mix may lead to the accumulation of voids at the interface [25]. Opaque thickness generally should not exceed 0.1 mm; otherwise, achieving an esthetic result, particularly in thin ceramic thicknesses, becomes impossible [26]. Increasing the opaque thickness may increase the graying effect of the opaque layers, which was attributed to the increase in the diffuse reflection properties of the opaque ceramic [26]. 

Moreover, the degree of compaction of the ceramic particles by the different technicians may be one of the manipulative variables affecting the ceramic [27]. The degree of compaction may also affect the amount of resultant porosity especially when improper powder/liquid ratio was used. The presence of porosities has always been a problem in the production of dental ceramic [27]. This not only resulted in undesirable roughness and pits when grinding the ceramic, but also exerts an even more undesirable effect on the strength and optical properties of the ceramic. These porosities may act as scattering centers for the light. With increasing the thickness of the veneering ceramic, the effect of porosities might be exaggerated. 

It was noticed that increasing the ceramic thickness by the technicians T1 and T2 increased the color difference (∆E) between the target shade (3M2) and the duplicated shade of the specimens, which may be due to poorer ceramic compaction for greater thicknesses. It was observed that these two technicians used a thin ceramic mix with excess water that may evaporate during the firing process, leaving voids acting as scattering centers with a pronounced negative effect at higher ceramic thicknesses [28]. While in the thinner ceramic thickness, the effect of these defects was not as exaggerated as that in thicker sections with accumulated voids.

Contrary to the specimens fabricated by the third technician, T3, which showed a decrease in ∆E values (better results) with increased ceramic thickness, this may be due to the proper condensation of ceramic powder, as it was observed that the third technician used thick mix consistency and did more compaction with minimal porosities. So, increasing the ceramic thickness gave better results.

Regarding the 1 mm ceramic thickness (th_1_), T_1_ and T2 showed lower ∆E than T_3_. This may be due to the thick mix of opaque layers, especially the wash-opaque used by T_3_, Figure 3. This thick mix resulted in increasing the thickness of the opaque layers by T_3_ (0.4 ± 0.05 mm) in comparison to those by T_1_ and T2 (0.1 ± 0.05 mm). This had been attributed to the increase in the diffuse reflection properties of the opaque ceramic ^(64)^ in T_3_th_1_ specimens. Thus, by reducing the ceramic thickness, the specimens became greatly affected by the graying effect of the opaque layer. 

On the other hand, increasing the thickness of the veneering ceramic of the specimens by T_3_ showed better results than T_1_ and T_2,_ with ceramic thickness of 1.5 mm and 2 mm (denoted by th_2_ and th_3,_ respectively). This may be attributed to the compaction technique with the least amount of porosity by T_3_ [29]. Thus, increasing the thickness of the enamel and dentin ceramics with good compaction gave low ∆E values and, at the same time, masked the opaque shade in T_3_th_2_ and T_3_th_3_ specimens [29]. 

Regarding the intra-technician variability, the color difference (∆E) values between the shades of the specimens duplicated by the same technician ranged between 0.2 and 1.5. These values may point to low intra-technician variability. These color differences were lower than the perceptibility threshold or just above it. This may indicate the intended, almost repeated manipulative procedures followed by each technician. This minor variation in repeating the exact shade with standardized ceramic thickness and technician may be attributed to the manipulative step that building both the dentin and the enamel ceramic together made it difficult to determine the thickness of each layer separately. Dental technicians could not create a predictable and exact thickness for each ceramic layer, which might hinder standardizing the layering pattern. 

However, dental technicians could be advised to reduce the use of unnecessary excess water or liquid in mixing and to perform proper ceramic compaction in order to minimize the possibility of porosities, which act as light scattering centers. In addition, reducing the thickness of the opaque layer could be beneficial, especially in thin ceramics, to avoid its unaesthetic, opaque optical characteristics.

Since the current study was limited to a small number of technicians and one ceramic shade, further research about the effect of ceramic manipulative variables is recommended for larger numbers of technicians and more ceramic shades. In addition, the microstructure of the resultant ceramic could be assessed microscopically.

## 5. Conclusions

Proper shade duplication in metal-ceramic restoration was a cumulative technique that was intimately related to the manipulative variables and to the ceramic thickness. Proper condensing of the ceramic powder and minimizing excess water gave better results with increased ceramic thickness. However, in a thinner ceramic section, increasing the opaque layer thickness may negatively affect the aesthetics even with proper condensation. Thus, the shade of ceramo-metallic restorations depended on the ceramic mix consistency, the opaque layer thickness, the degree of compaction, and the ceramic thickness.

## Figures and Tables

**Table 1:** Materials used in this study.

Material	Commercial Names	Composition (wt %)	Manufacturer
Veneering ceramic for metal substructures	Vita VM13	SiO_2_, Al_2_O_3_, K_2_O, Na_2_O, TiO_2_, CeO_2_, ZrO_2_, CaO, B_2_O_3_, BaO, SnO_2_, Mg-, Fe-, P-Ox	Vita, Zahnfabrik, Bad Säckingen Germany
Glazing materials	Vita Akzent Glaze	SiO_2_, Na_2_O, K_2_O, MgO, CaO, BaO, B_2_O_3_, Al_2_O_3_, Fe_2_O_3_, TiO_2_, P_2_O_5_, ZrO_2_, SnO_2_	Vita, Zahnfabrik, Bad Säckingen Germany
Nickel-chromium based dental alloy	System KN	Ni, Cr, Mo, Si	Adentatac, Köln, Germany

**Table 2:** Mean ΔE values between the target shade and those duplicated in different ceramic thicknesses by the different technicians (inter-technician variability).

∆E	Ceramic th1 (1 mm)	Ceramic th2 (1.5 mm)	Ceramic th3 (2 mm)	*p*-Value
Technician 1	2.1 a I ± 0.2	4.5 b II ± 0.4	6.4 c II ± 0.5	0.00001 *
Technician 2	1.8 a I ± 0.6	3.7 b II ± 1	5.1 c II ± 0.2	0.00001 *
Technician 3	3.4 c II ± 0.9	2.3 b I ± 0.2	2 a I ± 0.1	0.00001 *
	0.00001 *	0.00001 *	0.00001 *	

Means with different small letters in the same row show a significant difference, while means with different capital roman numbers in the same column also show a significant difference. * Indicates a statistically significant difference (*p*-value < 0.05).

**Table 3:** Mean ∆E values between the shades of the specimens duplicated by the same technician in thicknesses th1, th2, and th3 (intra-technician variability).

∆E	Ceramic th1 (1 mm)	Ceramic th2 (1.5 mm)	Ceramic th3 (2 mm)	*p*-Value
Technician 1	0.2 ± 0.1	0.6 ± 0.3	0.7 ± 0.5	0.8
Technician 2	0.8 ± 0.4	1.3 ± 0.6	1.5 ± 0.7	0.7
Technician 3	1.2 ± 0.7	0.3 ± 0.1	0.1 ± 0.05	0.2
	1	0.6	0.1	

The statistically significant difference level was set at a *p*-value < 0.05.

## Data Availability

The data presented in this study are available in this article.
